# Open access and predatory publishing: a survey of the publishing practices of academic pharmacists and nurses in the United States

**DOI:** 10.5195/jmla.2022.1377

**Published:** 2022-07-01

**Authors:** Bridget C. Conlogue, Neyda V. Gilman, Louisa M. Holmes

**Affiliations:** 1 bridget.conlogue@scranton.edu, Research & Scholarly Services, Weinberg Memorial Library, The University of Scranton, Scranton, PA; 2 ngilman@binghamton.edu, Associate Librarian, Assistant Head of Sustainability & STEM Engagement, Librarian for the Decker College of Nursing & Health Sciences and the School of Pharmacy & Pharmaceutical Sciences, Binghamton University, Binghamton, NY; 3 lmholmes@psu.edu, Assistant Professor, Geography & Demography, The Pennsylvania State University, University Park, PA

**Keywords:** Open access, predatory publishing, scholarly communication, academic publishing, tenure, pharmacists, nurses

## Abstract

**Objective:**

Academics are under great pressure to publish their research, the rewards for which are well known (tenure, promotion, grant funding, professional prestige). As open access publishing gains acceptance as a publishing option, researchers may choose a “predatory publisher.” The purpose of this study is to investigate the motivations and rationale of pharmacy and nursing academics in the United States to publish in open access journals that may be considered “predatory.”

**Methods:**

A 26-item questionnaire was programmed in Qualtrics and distributed electronically to approximately 4,500 academic pharmacists and nurses, 347 of whom completed questionnaires (~8%). Pairwise correlations were performed followed by a logistic regression to evaluate statistical associations between participant characteristics and whether participants had ever paid an article processing fee (APF).

**Results:**

Participants who had published more articles, were more familiar with predatory publishing, and who were more concerned about research metrics and tenure were more likely to have published in open access journals. Moderate to high institutional research intensity has an impact on the likelihood of publishing open access. The majority of participants who acknowledged they had published in a predatory journal took no action after realizing the journal was predatory and reported no negative impact on their career for having done so.

**Conclusion:**

The results of this study provide data and insight into publication decisions made by pharmacy and nursing academics. Gaining a better understanding of who publishes in predatory journals and why can help address the problems associated with predatory publishing at the root.

## INTRODUCTION

There is enormous pressure on members of the global academic community to publish research in scholarly journals. Publications, or lack thereof, may impact grant funding, tenure and promotion, awards, and institutional and professional reputation. Over the last twenty years, publishing alternatives to the traditional subscription-based journal model have continued to expand, one of which is open access (OA).

Open access as a movement was formally introduced in 2002 through the Budapest Open Access Initiative [1]. The initiative promoted scientific and other research as a public good that would harness the internet to make peer-reviewed journal literature freely available to all [1]. This basic definition of open access now includes a number of subtypes [2], the most relevant to our study being: green open access, which allows placement of the author manuscript in an institutional repository and which may require an embargo period; gold open access, in which authors retain copyright to the published article which is made freely available in the online journal; and hybrid open access, in which an article is open in a subscription-based journal. Publishing open access continues to gain traction as funders, including the U.S. National Institutes of Health and the Bill and Melinda Gates Foundation, require researchers to make their work freely available [2,3]. Perceived citation advantage, broader societal impact, dissemination of research due to social media tools, and altmetrics may also influence scholars' choice to publish open access [4]; open access enables scholars to have control over their work post-publication [5]. Antelman urges librarians to consider the impact of open access journals on collection development and their presence as part of the rapidly changing scholarly communications landscape [6].

To cover production and operating costs, publishers traditionally charge for journal subscriptions. In open access, these costs may be recouped by article processing fees (APF), which are paid by the authors(s). This arrangement in part has given rise to what are commonly referred to in the literature as “predatory publishers.” Masquerading as legitimate open access publishers, they present themselves as publishers of scholarly journals. It is estimated that around 70,000 articles in the biomedical literature were produced in 2014 in predatory journals [7].

Predatory publishing is a much-discussed phenomenon in scholarly literature [8–16]. There are many editorials and opinion pieces on the topic [17–20], and a number of studies [7,10,21–26] that investigate the motivations and rationale of academics in selecting these types of journals for publication of their work. There has been controversy surrounding use of the term “predatory,” what criteria identifies a publisher as such, and who has the authority to make such a determination [27,28]. Moher et al. assert it is generally assumed that engagement with predatory publishing is confined to Low and Middle Income Countries (LMICs) [10]. The studies referenced above provide data that academics in industrialized countries are indeed publishing in predatory journals. For example, one study examined 1,907 papers from journals known to be predatory, or likely predatory, and found that 15% of the corresponding authors of these papers were from the United States [10]. Data such as this led us to question why predatory journals were chosen. Were the authors duped, or did they make a conscious choice to publish in one of these journals?

To investigate these questions, we designed a study to gather information on the publishing decisions and practices of pharmacy and nursing academics based solely in the United States, with a focus on open access and predatory publishing. The study would allow authors to self-identify as having possibly published in a predatory journal, as opposed to finding and focusing on authors who have already published in known predatory journals, as was the approach in earlier studies [21,22,24,26]. Two of the authors of this study worked as health sciences librarians in support of the schools of pharmacy and nursing at their respective universities. We were often requested to evaluate open access journals, especially those promoted in direct emails to faculty, and we worked to steer faculty and students away from journals that may be predatory. Based on our experiences, and our reading of the literature, the survey was designed to explore the following four research questions: (1) how do academic pharmacists and nurses find and select journals to submit to?; (2) do they select open access journals for publication of their work?; (3) what are their experiences with predatory publishing; and (4) is level of experience or junior status a factor in these decisions?

## METHODS

### Study sample

The study was designed using nonprobability convenience sampling, recruiting participants from broad-reaching national listservs to which we had access. A 26-item questionnaire, programmed in Qualtrics, was distributed in February 2020 to 4,038 academic pharmacists via the Association of American Colleges of Pharmacy (AACP) Council of Faculty Forum (COF). The survey was also distributed to pharmacy and nursing contacts at a large healthcare system, to nursing and pharmacy faculty at our respective universities, and to various medical library-related listservs with a request to distribute to possible respondents. Nurses and pharmacists who received the survey directly from us were asked to share the survey with colleagues. The survey was re-distributed to AACP COF at the end of February, and again at the end of March for a total of 3 attempts to gain responses. The survey closed on April 13, 2020. The estimated total number of people who received the survey is approximately 4,500; from that 347 completed questionnaires were received (~8%) and 335 observations were retained for analysis. As the study was focused on predatory publishing in the US, we excluded 11 participants from the analyses who indicated that they resided in another country and one participant who was library staff and therefore not a target of the survey. The study was approved by the Wilkes University Institutional Review Board.

### Measures

For the purposes of this study, we crafted this definition of predatory publishing and included it in the survey: “[A]n entity that masquerades as a legitimate academic open access publisher of scholarly works. Questionable practices include, but are not limited to, non-existent peer review, false indexing claims, extremely rapid time to publication (‘extremely rapid' defined as publication within a few days to a couple of weeks, in spite of promised ‘peer review'), unexpected or unadvertised article processing fees, and false editorial boards.” Respondents were asked general demographic questions relating to whether they were based in a U.S. institution, their discipline, professional role, and faculty status, followed by a series of questions about why they published, how they evaluate their research impact, their familiarity with predatory publishing, and whether they had published in an open access journal, among other things. The full survey questionnaire can be found in the Appendix. Twelve respondents who were not based in a US institution were dropped from the sample. The measures used in the analysis were as follows:

“Article processing fee,” the dependent variable, was a binary variable indicating whether or not the participant had ever paid an APF.

“Position title” was a categorical variable indicating whether a participant was an Assistant, Associate or Full Professor, Lecturer/Researcher or instead worked for a Healthcare System.

“University/college research intensity” was a categorical variable indicating whether the participant's college or university was (1) low research-intensive; (2) moderate research-intensive; or (3) very research-intensive.

“Number of articles published” indicated how many peer-reviewed journal articles the participant had published; if the participant answered “0” they skipped to the end of the survey and were not included in further analyses.

**Figure 1:**
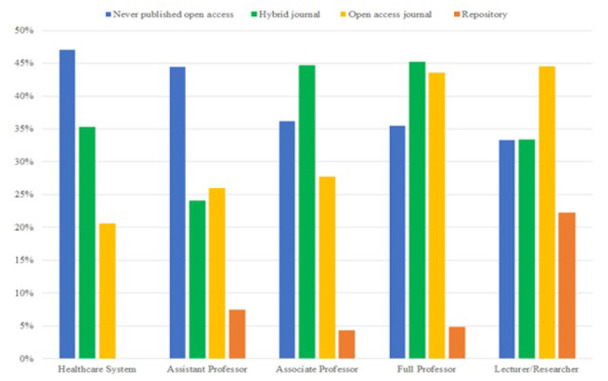
Participants' open access publishing experience, by position (N=335).

“Open access” was a binary variable, derived from a categorical variable, indicating whether participants had ever published in an open access journal (1) or not (0). The original set of responses included: (1) never published open access; (2) hybrid journal; (3) open access journal; and (4) repository (Figure 1).

*APRN = Advanced Practice Registered Nurse

“Familiarity with predatory publishing” was a categorical variable in response to the question “how familiar are you with ‘predatory publishing?’” Responses were (1) I have never heard this term; (2) I have heard the term but am unfamiliar with its meaning; (3) I have heard the term and am somewhat aware of the issues that surround it; (4) I would consider myself very knowledgeable on the topic.

“How do you decide which journal to submit to?” was a multiple-answer question including these two responses used in the analyses: (1) Resources like JCR, SJR, JANE; (2) Search on Google Scholar.

“I evaluate my research impact using the following” was also a multiple-answer question including these two responses used in the analyses (1) h-index; (2) Journal Impact Factor.

### Scale variables

Participants were also asked to respond to a series of questions and statements with Likert scale responses from 1-5 (low importance to high importance). The following questions and corresponding responses (for longer response lists, see Appendix) were included in our analyses:

“Journal metrics are important for…” (1) Tenure; (2) Professional reputation.

“My research metrics are important for…” (1) Tenure; (2) Professional reputation.

“Why do you publish?” (1) It is part of the scientific process; (2) I enjoy it.

### Statistical analysis

**Figure 2:**
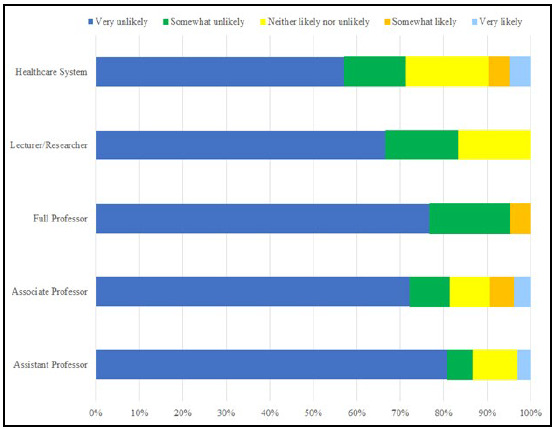
Responses to survey question: “What is the likelihood that any of your articles were published in a journal that is (possibly) predatory?” by Position Title (N=74)

The survey questionnaire included several skip patterns such that those included in the analysis were participants who had published at least one article, had published open access, had ever paid an APF, and thought it “very likely” to “somewhat unlikely” that they had ever “published in a journal that is (possibly) predatory,” leaving 183 out of the original 335 eligible participants. We used “ever paid an article processing fee” as the dependent variable because this is a potential indicator that a participant published in a predatory journal. Responses to this question are more reliable than asking directly about whether a participant published in a predatory journal because such journals are difficult to distinguish, especially for people newer to publishing. Below we report the likelihood that a participant paid an APF, controlling for a variety of characteristics. This does not demonstrate with any certainty that they published in a predatory journal, but it does offer insight into who is publishing open access and why. Descriptive statistics were calculated using Stata v16. We first conducted pairwise correlations between our dependent variable and the multiple answer variables to determine which responses were most associated with having paid an APF, the dependent variable. We then retained variables from each set of items that used Likert responses, had high correlations, and were theoretically appropriate to conduct logistic regression analyses, a technique that models the probability that something occurred (having paid an APF, for example), and addressed our research questions, stated above. Participants who worked in a healthcare system (n=18) were excluded from the regression analyses as their professional statuses do not align with academic ranks, nor are they concerned with academic tenure; they also were not asked about the level of research intensity at their institutions as these rankings are specific to academic institutions, so their inclusion would skew the results. There were also five participants who did not indicate a job title and were therefore excluded. This left 160 participants in the regression analyses. However, healthcare systems professionals are included in Figures 1, 2, and Table 2 to illustrate their potential to publish in predatory journals.

## RESULTS

### Demographics

Table 1 illustrates the demographic profiles of survey participants, organized by discipline. Pharmacy Practice was the largest discipline represented by far with 182 respondents, followed by Pharmaceutical Sciences with 45. Of the academic faculty members who responded, 32.3% were Assistant, 28.4% were Associate, 23.4% were Full Professors, and 6.8% were Lecturers/Researchers. Those in healthcare systems represented 9.1% of the overall sample. Participants were more likely to be from very research-intensive (44.0%) or low research-intensive (35.7%) institutions than those that were moderate research-intensive (20.3%). Thirty-five percent of participants had published 20 or more journal articles while 9.8% had not yet published any, primarily those in the Nursing professions. The survey was programmed to terminate the questionnaire for anyone who had not published any articles; they did not complete the questionnaire and were not included in any analyses.

**Table 1:** Survey participant demographic and publication characteristics, 2020 Binghamton-Wilkes University Predatory Publishing Survey (N=335).

	Nursing APRN	Nursing non APRN	Pharmacy Practice	Pharmacy Research	Pharmacy Administration	Pharmaceutical Science
N	24	33	182	25	26	45
Position Title						
Assistant Professor	26.3%	51.9%	40.1%	45.8%	12.0%	17.8%
Associate Professor	21.1%	29.6%	30.5%	16.7%	28.0%	44.4%
Full Professor	10.5%	14.8%	14.4%	25.0%	40.0%	35.6%
Adjunct Faculty	21.1%	3.7%	0.0%	4.2%	12.0%	0.0%
Health Systems	21.1%	0.0%	15.0%	8.3%	8.0%	2.2%
University/college research intensity						
Low research intensive	37.5%	12.5%	55.2%	29.4%	31.3%	48.3%
Moderate research intensive	12.5%	25.0%	20.9%	17.7%	25.0%	20.7%
Very research intensive	50.0%	62.5%	23.9%	52.9%	43.8%	31.0%
Number of articles published						
0	21.7%	25.0%	5.6%	4.0%	0.0%	2.2%
1–2	21.7%	15.6%	19.6%	8.0%	11.5%	0.0%
3–5	17.4%	21.9%	18.4%	8.0%	7.7%	2.2%
6–9	13.0%	21.9%	21.8%	8.0%	7.7%	8.9%
10–19	4.3%	6.3%	17.9%	16.0%	23.1%	31.1%
20 or more	21.7%	9.4%	16.8%	56.0%	50.0%	55.6%
Open access publishing						
Ever published open access	33.3%	28.1%	48.4%	72.0%	65.4%	63.6%

Figure 1 shows open access publishing trends by position. Nearly half of healthcare systems professionals (47.1%) reported that they had never published in open access journals compared to 33.3% of Lecturers/Researchers. Healthcare systems professionals did not use article repositories either, while a small percentage of faculty had done so. A much larger percentage of Lecturers/Researchers (44.4%) and Full Professors (43.5%) had published fully open access compared to other participants, while nearly half of Associate (44.7%) and Full Professors (45.2%) had published in a hybrid journal.

Table 2 illustrates how pharmacy and nursing professionals decide to which academic journals they submit their papers. Thirty participants did not indicate their job title; Table 2 includes the remaining 307 participants who did. Most participants endorsed journals whose scope best fit the topic under study while few utilized librarians or academic indexes. Assistant and Associate Professors were more likely to rely on colleague recommendations than Full Professors or Healthcare System professionals, while Full Professors were the most likely to use indexes, such as MEDLINE. Lecturers/Researchers were the most likely to submit to the journals they read.

**Table 2:** How participants decided on journals for article submission, by position title (N=307).

	Assistant Professor	Associate Professor	Full Professor	Lecturer/Researcher	Healthcare System*
	N				
How do you decide which journal to submit an article to?	108	94	62	9	34
Journals where scope fits with my topic	83.3%	96.8%	100.0%	77.8%	88.2%
Submit to the journals I read	58.3%	67.0%	67.7%	88.9%	52.9%
Colleague recommendations	56.5%	53.2%	32.3%	77.8%	41.2%
Check that it's on PubMed.gov	31.5%	27.7%	25.8%	22.2%	26.5%
Indexes including MEDLINE and Web of Science	17.6%	10.6%	21.0%	55.6%	2.9%
Resources such as JCR, SJR. JANE	13.0%	12.8%	11.3%	0.0%	2.9%
Google Scholar	7.4%	6.4%	6.5%	11.1%	**0.0%**
Librarian recommendations	5.6%	6.4%	4.8%	33.3%	2.9%

* Participants who worked for a healthcare system and did not indicate an academic/faculty appointment

Table 3 shows participant characteristics for the measures included in regression analyses, separated according to whether the participant had acknowledged publishing in an open access journal. Those at low or moderate research-intensive colleges or universities were less likely to have published in open access journals than those at high research-intensive institutions. Full Professors were the most likely faculty members to have published open access. Participants who had published more articles were more familiar with predatory publishing, and those who were more concerned about research metrics and tenure were also more likely to have published in open access journals. Those who indicated they published for enjoyment were less likely to have published open access, while those who regarded it as “part of the scientific process” were much more likely to have done so.

**Table 3:** Participant characteristics categorized by open access publishing record (N=160).

	Had not published open access		Published open access	
	μ or %	S.D.	μ or %	S.D.
	N=90		N=70	
University research focus				
Low research intensive	51.1%	0.50	34.3%	0.48
Moderate research intensive	23.3%	0.43	22.9%	0.42
High research intensive	25.6%	0.44	42.9%	0.50
Position title				
Assistant professor	28.9%	0.46	28.6%	0.46
Associate professor	38.9%	0.49	28.6%	0.46
Full professor	20.0%	0.40	31.4%	0.47
Lecturer/Researcher	12.2%	0.33	11.4%	0.32
Number of articles published (1–5 scale)	3.37	1.35	4.31	0.97
Familiarity with predatory publishing (1–4 scale)	2.82	0.91	3.27	0.78
How do you decide to which journal to submit?				
Resources like JCR, SJR, JANE	8.9%	0.29	17.1%	0.38
Search on Google Scholar	6.7%	0.25	12.9%	0.34
I evaluate my research impact using the following:				
h-index	22.2%	0.42	52.9%	0.50
Journal Impact Factor	38.9%	0.49	52.9%	0.50
Journal metrics are important for:				
Tenure (1–5 scale)	3.60	1.20	3.92	1.13
Professional reputation (1–5 scale)	4.15	0.83	4.06	0.93
My research metrics are important for:				
Tenure (1–5 scale)	3.27	1.29	3.86	1.17
Professional reputation (1–5 scale)	4.02	1.04	3.99	1.00
Why do you publish?				
It is part of the scientific process	57.8%	0.50	90.0%	0.30
I enjoy it	62.2%	0.49	64.3%	0.48

AOR = Adjusted odds ratio; C.I. = confidence interval; *p < .05; **p<.01; ***p<.001

Table 4 shows logistic regression results for having paid an APF. Participants who had published more articles were more likely to have published open access (AOR=1.67). Participants who endorsed “resources like JCR, SJR, JANE” for journal decision-making were more than four times more likely to have paid an APF than other participants, and those who used the h-index to evaluate their research impact had nearly three times higher odds of having paid an APF. Participants who indicated that “my research impact metrics are important for tenure” were 83% more likely to have paid an APF, while those who valued research metrics as important for professional reputation were 55% less likely to have paid an APF. Finally, participants who published because “it is part of the scientific process” were seven times more likely to have paid an APF than those who did not choose this option, while those who published because they “enjoyed it” were 64% less likely to have paid an APF.

**Table 4:** Logistic regression results of article processing fees on participant characteristics and opinions (N=160).

	AOR	C.I.
	N=160	
University research focus		
Low research intensive (referent)		
Moderate research intensive	0.46	[0.15, 1.43]
High research intensive	0.87	[0.31, 2.46]
Position title		
Full professor (referent)		
Assistant professor	1.00	[0.28, 3.58]
Associate professor	0.43	[0.14, 1.34]
Lecturer/Researcher	1.77	[0.39, 7.99]
Number of articles published	1.67	[1.05, 2.68] *
Familiarity with predatory publishing	1.27	[0.73, 2.20]
How do you decide to which journal to submit?		
Resources like JCR, SJR, JANE	4.35	[1.30, 14.51] *
Search on Google Scholar	2.14	[0.55, 8.31]
I evaluate my research impact using the following:		
h-index	2.62	[1.06, 6.51] *
Journal Impact Factor	1.43	[0.61, 3.35]
Journal metrics are important for:		
Tenure	0.84	[0.55, 1.27]
Professional reputation	1.33	[0.66, 2.68]
My research metrics are important for:		
Tenure	1.83	[1.15, 2.93] *
Professional reputation	0.45	[0.23, 0.86] *
Why do you publish?		
It is part of the scientific process	7.03	[2.19, 22.61] **
I enjoy it	0.36	[0.14, 0.96] *

AOR = Adjusted odds ratio; C.I. = confidence interval

* p < .05

** p<.01

*** p<.001

### Awareness of Predatory Publishing

A Likert scale response was used to measure awareness of predatory publishing. The majority of respondents (53.44%) selected “I have heard the term and am somewhat aware of the issues that surround it.”63 (20.66%) consider themselves “very knowledgeable on the topic” while 48 respondents (15.74%) indicated “I have never heard this term.” All participants who indicated they were familiar with the term were presented with choices to indicate how they had learned about predatory publishing with the majority (45.81%), indicating they had learned about it from reading the literature and from colleagues (64.2%). Other means of learning about predatory publishing included a program or information session (22.18%), from a librarian (24.12%), through email solicitations from predatory publishers (3.89%), and firsthand experience (2.33%).

The majority of respondents “somewhat agree” (40.98%) that they cite only journals they know and trust. Ninety-six respondents (31.48%) “strongly agree” with this statement. The responses to “My institution or department offers assistance or advice about what journals to publish in” was fairly evenly distributed between “somewhat agree” (19.34%), “neither agree nor disagree” (23.93%), “somewhat disagree” (20.98%), and “strongly disagree” (22.30%).

Figure 2 illustrates how likely the participants who responded yes to the question asking whether they had ever paid an APF thought it was that they had published in a predatory journal. No Full Professors or Lecturers/Researchers reported that they were “very likely” to have done so, but 4.8% of Healthcare Systems professionals, 3.7% of Associate Professors, and 2.9% of Assistant Professors acknowledged that they very likely had published in a predatory journal. Overall, Healthcare Systems professionals showed the greatest likelihood of engaging in predatory publishing, followed by Associate Professors. Alternatively, 80.1% of Assistant Professors reported that it was “very unlikely” they had published in predatory journals.

Respondents who selected “very unlikely” that they had published in a predatory journal were sent to the end of the survey. The remaining 29 respondents selected a varied number of reasons why there may be a chance a chosen journal could be predatory. The top reasons were lack of peer-review report (10.34%), too quick of a publishing turnaround (37.93%), lack of editing (20.69%), high or multiple fees (34.38%), unexpected fees (24.14%), publisher refused to withdraw article pre-publication (3.45%), and aggressive communication about payment of fee(s) for publication (10.34%).

When asked why they and/or their co-authors submitted to a journal that may have been predatory, 31.03% indicated it had studies similar to theirs, 68.97% indicated the journal scope was related to their area of research, 10.34% indicated a colleague had published in the journal, 34.48% indicated their article had been rejected by another publisher, 6.9% indicated pressure due to tenure/promotion portfolio deadline, 3.45% indicated that authors they admired had published in the journal, and 17.24% indicated that the primary author encouraged them to publish in the journal.

Participants were asked what they did when they realized the publisher was predatory. 58.62% took no action, 13.79% attempted to retract the article, 6.9% refused to pay the APF, 6.9% republished with a reputable journal, and 13.79% took other actions including writing editorials and negotiating the APF.

When asked about the potential impact of publishing in a predatory journal, 13.79% thought there could be a negative impact on tenure or promotion, grant applications (17.24%), and departmental reputation (20.69%), and 13.79% believed it would negatively impact job applications.

## DISCUSSION

The initial goals of this study were to explore awareness of open access, predatory publishing, and the factors that might influence nursing and pharmacy academics to publish in a potentially predatory journal. However, as some researchers may not want to admit to having published in a possibly predatory publication, even in an anonymous survey, and some researchers may not be fully aware of predatory publishing, broad questions about publishing were included. These potential biases are why the logistic regression model was run using data from the question on paying APFs, rather than the question on publishing in a predatory journal. While APFs are common in many non-predatory open access and hybrid open access journals, predatory journals almost always have an APF. Although running the regression model this way means the data is not solely focused on known predatory publications, it ideally results in a less biased overview compared to looking at data from self-acknowledged authors of articles in predatory journals. The distinction must be made that not all open access journals charge an APF and many that do are legitimate journals and are not predatory. Good quality subscription and open access journals may also charge fees for color or high page counts. Many provide thorough peer-review and editorial oversight, regardless of whether a fee is charged for publication. An APF (or lack of it), is not a single quality indicator for a journal. However, as the great majority of predatory publishers style themselves as open access and levy APFs, the amount of which can vary greatly [7,16], it is crucial to stress that while the regression model was done on the payment of APFs, not all APFs are indicative of predatory publishing.

Looking at the relationship between metrics and where individuals publish produced some results worthy of note. Respondents who indicated they see a tie between personal research impact metrics and professional reputation were less likely than others to pay an APF. Conversely, those who see a tie between their research impact metrics and tenure, were more likely to pay an APF. Interestingly, those who pay attention to h-indices are more likely to pay an APF. Since h-indices are purely citation based, these authors might place emphasis on the wide distribution for their work, with easier access and visibility that enables greater sharing and citing. Publishing in open access journals may lead to increased citations [29,30] and those who know about and use h-indices may be aware of this and may seek out open access journals to publish in. Similarly, that those who utilize resources such as JCR, SJR, or JANE are more likely to pay an APF could indicate that they are comfortable with those APFs because they have checked the validity of the journal(s) of interest.

Those who see publishing as a part of the scientific process are more likely to pay an APF while those who publish for personal enjoyment are less likely to do so. Those in the former group may experience more pressure to publish. They may be more likely to pay an APF because they know which APF-charging journals are legitimate.

The results looking at APFs provide data to discuss, but the nature of APFs, open access, and predatory publishing makes it difficult to determine exactly who is publishing in predatory journals and why. Pride in one's work, combined with the pressure to publish, and to publish in the top journals, creates challenges for discussions about predatory publishing, even in an anonymous setting. The last section of this survey asked participants if it was possible they had submitted to a predatory journal. Again, knowing for sure if a journal is predatory can be difficult, and there are differing degrees of “predatoriness.” It was only in 2019 that a consensus definition for predatory publishing was established [28]. Due to these limitations, the question focused on the possibility of publishing predatory rather than a more direct “did you or did you not” type of question. Even with this flexibility, some individuals adopted a defensive tone in the open comments section and some skipped follow-up questions and made statements that they never published predatory and never would. Reactions such as these are one of the main reasons questions in the survey focused on APFs, and offer an explanation as to why finding out more about those who have published in predatory journals is so difficult.

How respondents find journals to publish in was another topic covered by the survey. One of the first questions asked was how decisions were made on what journals to publish in. Respondents who later indicated they may have submitted to a possibly predatory journal were asked a similar question about why they decided to submit to that particular journal. Most respondents to the first general question identified journal scope as the reason, and the second most frequent reason was that their article had been rejected by other publishers. To the latter point, a respondent made this comment at the end of the survey:

We didn't have great data and wanted to focus more on the implementation aspect of our intervention. We knew it would be difficult to publish in a more reputable journal.

Although their manuscript had not yet been rejected by any publisher, the commenter acknowledges publishing predatory as an outlet for publishing lesser quality work, and that the ends justifies the means.

The majority of respondents in both pools noted that colleague recommendations are a major way of identifying journals, with the second and third most selected reasons being (1) submitting to the journals they read and (2) journal scope, respectively. However, for those responding to the questions focused on potentially predatory journals, email solicitations were identified as the most common way respondents learned about the journal. These emails often use flattery to encourage readers to submit their work and seem to offer an attractive path to publishing. These emails may be confused with calls for submissions from legitimate journals.

Of the participants who indicated there was a chance they submitted at least one paper to a possibly predatory publisher, the majority responded that they took no action upon considering the publisher may be predatory. This could be due in part to a number of individuals who, despite responding that they may have published in a predatory journal, believed the chance of this was so low it was not worth further investigation. It may also be that the possibility of publishing predatory was only considered upon reading the survey question and the respondents only answered in the affirmative because they could not answer in the negative with complete certainty. Other respondents who took no action may have experienced multiple rejections of a manuscript, felt relief when at last it was published somewhere, and pursued it no further. Of the respondents who did act, one commented that they wrote an editorial about their experience. Another respondent stated that they renegotiated the APF; while a lesser fee is commendable, it does not address the greater problems inherent in predatory publishing.

It was surprising that in response to the question whether publishing in a predatory journal had a negative impact on their career, most respondents indicated there was not much of an impact at all. The pressure to publish may override other concerns. On this point, a participant observed:

I think the culture within academics has fueled the need for low-level research and publishing of articles that lack substance. It also fuels predatory/questionable journals because people feel compelled to just get something out there so they do it through these means.

Some academics may gamble that quality will not be an issue or will not be noticed during the advancement process [21]. One participant commented:

In academe, publishing (in any form) is a ‘numbers game', i.e., quantity. Quality has little/less impact during evaluation/review processes. Hence, a significant basis for the statement, ‘Publish or perish.'

This may explain the lack of action upon the realization by those who indicated they had or may have published in a possibly predatory journal; unfortunately, the system of rewards at universities inadvertently may help fuel predatory publishing.

## STUDY LIMITATIONS

### Self-report survey design

The majority of the study's limitations are inherent to the study design of a survey instrument, including self-report bias. Great care was put into the language of the survey, but participants' bias for or against open access as a publishing practice may have impacted response rates.

Other limitations include an overall small sample size, especially when reduced to those who admitted to the possibility of publishing in a predatory journal. Initial calls for participants included posting on the AACP COF community page. Second and third calls went to the group via individual emails as in March 2020 posting surveys to the community pages was no longer permitted. It is unknown how this change affected the sample size. As two of us were members of AACP, reaching a large number of academic pharmacists was fairly straightforward. To reach the academic nursing population, we made use of individual connections, resulting in less than a 17% response rate from nursing. We made arrangements to distribute the survey to nursing forums, but due to the pandemic and other circumstances this did not happen as planned. To our knowledge, a nursing equivalent to AACP does not exist, i.e. an organization that includes librarians/information professionals.

## CONCLUSION

This study provides insight into publication decisions made by health professions academics in the United States. It contributes information about awareness of and engagement with open access and predatory publishing in an industrialized country and highlights the complex issues surrounding academic research, publishing, and systems of rewards. Gaining a better understanding of who publishes in predatory journals, and the reasons for doing this can help administrators, department chairs, faculty mentors, and librarians in raising awareness at their institutions and addressing the problem at the root. This is especially urgent in the health sciences as healthcare practitioners rely on the primary literature to inform patient care [9, 12, 15, 31, 32]. Articles published in predatory journals have been included in systematic reviews, which are high-level evidence syntheses often used in clinical decision making [33]. Librarians on systematic review teams, and those who conduct literature searches for clinical faculty should be on the alert for articles from journals that may be predatory; those who are involved in instruction and scholarly communication should take every opportunity to educate faculty, clinicians, and students about predatory publishing, and raise awareness of its potential damaging effects on the clinical information ecosystem [10]. Determining whether an open access journal is predatory is reliant on multiple factors; librarians bring to this conversation their expertise in the evaluation of information sources. Academic publishing in general has many issues, is complex, and is continuing to change. Many of these changes involve open access, and education about the concept and about predatory publishing is key. Specifically, differentiation needs to be made and predatory journals avoided.

More research is needed, and in order for that research to provide significant findings, frank and open discussions about predatory publishing where authors do not feel under attack are necessary.

## Data Availability

Data associated with this article are available in the Open Science Framework at: https://osf.io/9hvje/?view_only=f98e8a2a5b334e22a57bacf8ba0338b0.

## SUPPLEMENTAL FILES

**Appendix A:** Wilkes-Binghamton Faculty Survey
