# Supplementary material for: Open access and predatory publishing: a survey of the publishing practices of academic pharmacists and nurses in the United States
Source: J Med Libr Assoc. 2022 Jul 1;110(3):294–305. doi: 10.5195/jmla.2022.1377 (PMC9782588; doi:10.5195/jmla.2022.1377)
Supplement: Supplementary file 1 — Appendix A: Wilkes-Binghamton Faculty Survey [file jmla-110-3-294-s01.pdf]

Appendix A: Wilkes-Binghamton Faculty Survey

Start of Block: CONSENT

Health Sciences Librarians from Wilkes University and the State University of New York (SUNY), Binghamton wish to explore the publishing practices of pharmacy and nursing faculty and clinical practitioners at U.S. institutions as well as their viewpoints on the current landscape of academic publishing, with a focus on Open Access publishing. We are focusing on Pharmacy and Nursing as we support faculty in these disciplines as librarians at our respective universities. We would appreciate your responses to this voluntary survey. The survey will take about 10-15 minutes to complete. Your responses will be recorded anonymously. Any data that could possibly be associated with you or your institution will be de-identified. There are no potential risks for participating in this study, which has received approval from the Wilkes University IRB in cooperation with SUNY Binghamton IRB. The data from the survey may be shared either at a national conference in a poster or in a published journal article; this data may not benefit you directly but may contribute to the larger conversation on scholarly publishing. There are questions in the survey on the topic of predatory publishing. For the purposes of this survey, a predatory publisher (or standalone predatory journal) is defined as an entity that masquerades as a legitimate academic Open Access publisher of scholarly works. Questionable practices include, but are not limited to, non-existent peer review, false indexing claims, extremely rapid time to publication ("extremely rapid" defined as publication within a few days to a couple of weeks, in spite of promised "peer review"), unexpected or unadvertised article processing fees, and false editorial boards. **By selecting "Yes" to the first question in this survey you are consenting to participate in this study.** You may stop taking the survey at any time. You may refuse to answer any question in the survey. For questions or concerns, please contact the investigators for this study:
Bridget Conlogue.
bridget.conlogue@wilkes.edu (570-408-4959)
Neyda Gilman. ngilman@binghamton.edu (607-777-4923)
If you have questions or concerns about your rights as a research participant, you may contact Wilkes University IRB: irb@wilkes.edu. Reference: Protocol #81

Q1 Would you like to consent to participate in this study?

○ Yes (1)

○ No (0)

End of Block: CONSENT

Start of Block: QUESTIONNAIRE

X→

Q2 Are you based in a U.S. institution?

◯ Yes (1)

◯ No (0)

Q3 What is your discipline?

▼ Nursing (APRN) (1) ... Other (7)

Display This Question:

If What is your discipline? = Other

Q4 What is your discipline?

____

Display This Question:

If What is your discipline? = Nursing (APRN)

Q4.1 What is your area of focus?

◯ Clinical practice/provider (1)

◯ Administration (2)

◯ Research (3)

Display This Question:

If What is your discipline? = Nursing (non-APRN)

Q4.2 What is your area of focus?

- ○ Clinical (1)
- ○ Administration (2)
- ○ Research (3)

Q5 My place of employment is a...(select all that apply):

- ☐ Community college (1)
- ☐ 4-year college (2)
- ☐ 4-year university (3)
- ☐ Research university (4)
- ☐ Teaching college/university (5)
- ☐ Public university (6)
- ☐ Private university (7)
- ☐ For-profit university (8)
- ☐ Healthcare system (9)
- ☐ Other (10)

Display This Question:

If My place of employment is a...(select all that apply): = Other

Q5.1 How else would you classify your place of employment?

____

Display This Question:

If My place of employment is a...(select all that apply): != Healthcare system

Q5.2 What is your current job title?

- ◯ Assistant Professor (1)
- ◯ Associate Professor (2)
- ◯ Full Professor (3)
- ◯ Lecturer (4)
- ◯ PhD/PharmD candidate (5)
- ◯ Researcher (6)
- ◯ Other (7)

Display This Question:

If What is your current job title? = Other

Q5.3 What is your job title?

____

Display This Question:

If My place of employment is a...(select all that apply): != Healthcare system

Q5.4 My university/college is:

- ◯ Very research intensive (e.g. designated High Research Activity) (1)
- ◯ Moderate research intensive (2)
- ◯ Low research intensive (e.g. a teaching-focused university) (3)
- ◯ Don't know (4)

Display This Question:

If My place of employment is a...(select all that apply): = Healthcare system

Q5.5 What is your current role (select all that apply)?

- ▢ Researcher (1)
- ▢ Clinician (Advanced Practice Nurse or Clinical Pharmacist) (2)
- ▢ Prescribing Provider (Pharmacy) (3)
- ▢ Postdoctoral/Resident (4)
- ▢ Administration (5)
- ▢ Other (6)

Display This Question:

If What is your current role (select all that apply)? = Other

Q5.6 What is your role?

____

Q6 How many total research articles have you published?
Count articles where you were one of 15 or fewer authors.

▼ 0 (0) ... 20 or more (5)

Skip To: End of Block If How many total research articles have you published? Count articles where you were one of 15 or f... = 0

Q7 How do you decide which journal to submit an article to?
Select all that apply.

- ▢ I submit to the journals I read (1)
- ▢ I look for journals in which the scope fits with my topic (2)
- ▢ Colleague recommendations (3)
- ▢ Librarian recommendations (4)
- ▢ I look in resources such as JCR, SJR, JANE, etc. (5)
- ▢ I check indexes including MEDLINE and Web of Science (6)
- ▢ I check that it is on PubMed.gov (7)
- ▢ I search on Google Scholar (8)
- ▢ Other (9)

Display This Question:

If How do you decide which journal to submit an article to? Select all that apply. = Other

Q7.1 What other way(s) do you decide which journal to submit articles to?

____

Q8 What impact metrics do you consider when choosing a journal to submit to? Select all that apply.

- ☐ Impact factor (Web of Science, Journal Citation Reports) (1)
- ☐ SJR (Scimago, Scientific Journal Rankings) (2)
- ☐ Eigenfactor (3)
- ☐ Elsevier CiteScore (4)
- ☐ None (5)
- ☐ Other (6)

Display This Question:

If What impact metrics do you consider when choosing a journal to submit to? Select all that apply. = Other

Q8.1 What other impact metric(s) do you consider?

____

Q9 I evaluate my research impact using the following:
Select all that apply.

- ☐ H-index (1)
- ☐ Altmetrics (2)
- ☐ Google Scholar (3)
- ☐ Impact Factor of journals in which I've published (4)
- ☐ Citation counts (5)
- ☐ I don't evaluate my own research impact (6)
- ☐ Other (7)

Display This Question:

If I evaluate my research impact using the following: Select all that apply. = Other

Q9.1 What other ways do you evaluate your research impact?

____

Q10 Journal metrics are important for:

	1 (low importance) (1)	(2)	(3)	(4)	5 (high importance) (5)	Not applicable (97)
Tenure (1)	○	○	○	○	○	○
Promotion (2)	○	○	○	○	○	○
Grant funding (3)	○	○	○	○	○	○
Professional development funds (4)	○	○	○	○	○	○
Professional reputation (5)	○	○	○	○	○	○

Q11 My research metrics are important for:

	1 (low importance) (1)	(2)	(3)	(4)	5 (high importance) (5)	Not applicable (97)
Tenure (1)	○	○	○	○	○	○
Promotion (2)	○	○	○	○	○	○
Grant funding (3)	○	○	○	○	○	○
Professional development funds (4)	○	○	○	○	○	○
Professional reputation (5)	○	○	○	○	○	○

Q12 Why do you publish?
Select all that apply.

- ▢ For tenure/promotion/job requirement (1)
- ▢ I want to share the results of my work (2)
- ▢ It is part of the scientific process (3)
- ▢ I enjoy it (4)
- ▢ It helps build prestige and reputation among my colleagues/research community (5)
- ▢ Other (6)

Display This Question:
If Why do you publish? Select all that apply. = Other

Q12.1 For what other reason(s) do you publish?

____

____

____

____

____

Q13 How familiar are you with "predatory publishing?"

- ◯ I have never heard this term (1)
- ◯ I have heard the term but am unfamiliar with its meaning (2)
- ◯ I have heard the term and am somewhat aware of the issues that surround it (3)
- ◯ I would consider myself very knowledgeable on the topic (4)

Display This Question:

If How familiar are you with "predatory publishing?" != I have never heard this term

Q14 How did you learn about predatory publishing?
Select all that apply.

- ▢ Reading the literature (1)
- ▢ From colleagues (2)
- ▢ At a program or information session (3)
- ▢ From a librarian (4)
- ▢ Other (5)

Display This Question:

If How did you learn about predatory publishing? Select all that apply. = Other

Q14.1 From what other source did you learn about predatory publishing?

____

Q15 Indicate to what extent you agree or disagree with the following two questions.

	Strongly agree (1)	Somewhat agree (2)	Neither agree nor disagree (3)	Somewhat disagree (4)	Strongly disagree (5)
I only cite journals I know and trust. (1)	o	o	o	o	o
My institution or department offers assistance or advice about what journals to publish in. (2)	o	o	o	o	o

Q16 Have you ever published an Open Access (OA) article?
Select all that apply.

▢ Yes, in a hybrid journal (has both OA and traditional articles) (1)

▢ Yes, in an OA journal (2)

▢ Yes, in a repository (3)

▢ No (4)

Display This Question:

If Have you ever published an Open Access (OA) article? Select all that apply. != No

X→

Q16.1 Have you ever paid an article processing fee?

- ◯ Yes (1)
- ◯ No (0)

Skip To: End of Block If Have you ever paid an article processing fee? = No

Q17 What is the likelihood that any of your articles were published in a journal that is (possibly) predatory?

- ◯ Very likely (1)
- ◯ Somewhat likely (2)
- ◯ Neither likely nor unlikely (3)
- ◯ Somewhat unlikely (4)
- ◯ Very unlikely (5)

Skip To: End of Block If What is the likelihood that any of your articles were published in a journal that is (possibly) p... = Very unlikely

Q18 How many of your articles have been published in a journal that may be predatory?

- ◯ 1 (1)
- ◯ 2-3 (2)
- ◯ 4-5 (3)
- ◯ More than 5 (4)

Q19 What was your authorship role on this/these paper(s)?
Select all that apply.

- ☐ First author (1)
- ☐ Last author (2)
- ☐ Senior author (3)
- ☐ Corresponding author (4)
- ☐ Contributing author (5)

Q20 Why do you think the journal(s) might have been predatory?
Select all that apply.

- ☐ Lack of peer review report (1)
- ☐ Too quick of a turnaround (2)
- ☐ Lack of editing (3)
- ☐ High/multiple fees (4)
- ☐ Unexpected fees (5)
- ☐ Publisher refused to withdraw article pre-publication (6)
- ☐ Aggressive communication about payment of fee(s) for publication (7)
- ☐ Other (8)

Display This Question:

If Why do you think the journal(s) might have been predatory? Select all that apply. = Other

Q20.1 What is the other reason you think the journal may have been predatory?

____

Q21 Where did you learn about this/these journal(s)?
Select all that apply.

▢ Email solicitation from publisher (1)

▢ From a colleague/advisor (2)

▢ Article reference list (3)

▢ Other (4)

Display This Question:

If Where did you learn about this/these journal(s)? Select all that apply. = Other

Q21.1 Where else did you learn about this/these journal(s)?

____

Q22 When in the process did you and/or your co-author(s) begin to suspect the journal?

○ Before submitting (1)

○ During the submission process (2)

○ After submitting (3)

○ I don't know (4)

Q23 What did you and/or your co-author(s) do when you realized the publisher was possibly predatory?
Select all that apply.

- ▢ Attempted to retract the article (1)
- ▢ Refused to pay the Article Processing Fee (2)
- ▢ Republished the paper with a reputable journal (3)
- ▢ Nothing (4)
- ▢ Other (5)

Display This Question:

If What did you and/or your co-author(s) do when you realized the publisher was possibly predatory?... = Other

Q23.1 What else did you do when you realized the publisher was possibly predatory?

____

Q24 Why did you and/or your co-author(s) decide to submit to the selected journal? Select all that apply.

- ▢ It had studies similar to mine (1)
- ▢ The journal scope was related to my (our) area of research (2)
- ▢ Colleagues had published in the journal (3)
- ▢ Publication turnaround time (4)
- ▢ My (our) article had been rejected by other publishers (5)
- ▢ Tenure/promotion portfolio deadline/pressure (6)
- ▢ Authors whose research I (we) admire were published in the journal (7)
- ▢ The primary author encouraged me (us) to publish in the journal (8)
- ▢ Other (9)

Display This Question:

If Why did you and/or your co-author(s) decide to submit to the selected journal? Select all that a... = Other

Q24.1 For what other reason did you decide to submit to the selected journal?

____

Q25 What impact do you feel publishing in a predatory journal may have had on the following?

	No impact (0)	Very negative (1)	Slightly negative (2)	Neither negative nor positive (3)	Slightly positive (4)	Very positive (5)
Rank and Tenure portfolio (1)	○	○	○	○	○	○
Grant applications (2)	○	○	○	○	○	○
Departmental/professional reputation (3)	○	○	○	○	○	○
Job applications (4)	○	○	○	○	○	○

End of Block: QUESTIONNAIRE

Start of Block: ADDITIONAL COMMENTS

Q26 Please offer any additional thoughts or comments about Open Access publishing, predatory or questionable journals, and/or publishing in general, or anything that you would like to add to your previous answers.

____

____

____

____

____

End of Block: ADDITIONAL COMMENTS
